# PrEP use and unmet PrEP-need among men who have sex with men in London prior to the implementation of a national PrEP programme, a cross-sectional study from June to August 2019

**DOI:** 10.1186/s12889-022-13425-0

**Published:** 2022-06-03

**Authors:** Dana Ogaz, Louise Logan, Tyrone J. Curtis, Lorraine McDonagh, Luis Guerra, Daniel Bradshaw, Poorvi Patel, Chiara Macri, Gary Murphy, O. Noel Gill, Anne M. Johnson, Anthony Nardone, Fiona Burns

**Affiliations:** 1 Blood Safety, Hepatitis, STI and HIV Division, Public Health England, London, UK; 2 Sexual Health, Reproductive Health and HIV, Public Health England, London, UK; 3 Institute for Global Health, University College London, London, UK; 4 National Institute for Health Research Health Protection Research Unit in Blood Borne and Sexually Transmitted Infections, University College London, London, UK; 5 Research Department of Primary Care and Population Health, University College London, London, UK; 6 National Infection Service Laboratories, Public Health England, London, UK; 7 Epiconcept, Paris, France; 8 Royal Free London NHS Foundation Trust, London, UK

**Keywords:** Men who have sex with men, MSM, Gay, Bisexual, HIV pre-exposure prophylaxis, PrEP, HIV

## Abstract

**Background:**

Access to prevention options, including HIV pre-exposure prophylaxis (PrEP), remains a public health priority for gay, bisexual, and other men who have sex with men (MSM), especially in London. We describe PrEP use in a London community sample of MSM before the introduction of a national PrEP programme in October 2020.

**Methods:**

From June–August 2019, MSM aged ≥ 18 recruited from London commercial venues were asked to self-complete a sexual health questionnaire and provide an oral fluid sample for anonymous HIV antibody testing. Descriptive analyses of demographic characteristics, service engagement and outcomes, as well as sexual risk and prevention behaviours were examined in the survey population and in those reporting current PrEP use. We performed sequential, multivariate analyses examining current PrEP use in MSM of self-perceived HIV-negative/unknown status with identified PrEP-need defined as the report of condomless anal sex (CAS) in the last three months, or the report of CAS (in the last year) with an HIV-positive/unknown status partner not known to be on HIV treatment, in reflection of UK PrEP guidelines.

**Results:**

One thousand five hundred and thirty-fifth questionnaires were completed across 34 venues, where 1408 were analysed. One in five MSM of self-perceived HIV-negative/unknown status reported current PrEP use (19.7%, 242/1230). In men with PrEP-need, 68.2% (431/632) did not report current use. Current PrEP use was associated with age (aOR: 3.52, 95% CI: 1.76–7.02 in men aged 40–44 vs men aged 18–25) and education (aOR: 1.72, 95% CI: 1.01–2.92 in men with ≥ 2 years/still full-time vs no/ < 2 years of education since age 16).

**Conclusion:**

Among MSM in London, PrEP use is high but there is indication of unmet PrEP-need in men of younger age and lower levels of post-16 education. National programme monitoring and evaluation will require continued community monitoring to guide interventions ensuring equitable PrEP access and uptake in those who could most benefit from PrEP.

**Supplementary Information:**

The online version contains supplementary material available at 10.1186/s12889-022-13425-0.

## Introduction

In England, and especially London, HIV prevention in gay, bisexual, and other men who have sex with men (MSM) remains a public health priority. With the implementation and scale-up of HIV combination prevention, including increased repeat HIV testing, treatment as prevention (TasP), condom use, and greater HIV pre-exposure prophylaxis (PrEP) availability, new HIV diagnoses in England, largely driven by London, are at a twenty-year low [1]. Numbers of sexually transmitted infections (STIs) in MSM, however, continue to increase to new highs [2]. Given pledged government commitment in 2020 [3], ending new HIV transmissions in England by 2030 may be achievable as PrEP implementation continues. The effect of PrEP on HIV incidence is so far unclear; recent modelling suggests [4] HIV incidence falls were driven by combination prevention in absence of widespread PrEP availability.

Adherent daily or event-based use of HIV PrEP is a highly effective HIV prevention option for MSM [5–7], however, access and roll-out across Europe has been varied [8]. PrEP availability across the UK began with online purchase from 2015 and programme availability in Scotland and Wales from 2017. In England, the PrEP Impact Trial was launched across 157 sexual health clinics (SHCs) in October 2017 to provide insights to outstanding implementation questions surrounding eligibility, uptake, and duration of PrEP use to inform future programme commissioning. Early recruitment was rapid and quickly met initial PrEP-need estimates of 10,000 MSM participants [9], prompting two trial expansions through its enrolment duration to July 2020. Further reduction of generic PrEP costs fuelled online availability and affordability of private PrEP, where community groups and outreach facilitated procurement for men seeking PrEP outside of available trial sources [10]. During the summer of 2019, recruitment to the PrEP Impact Trial was marred by lengthy recruitment pauses for MSM following rapid enrolment, especially in London SHCs, triggering the doubling of available trial places from 13,000 to 26,000 [11] to enable accurate trial objective analysis.

Implementation of an uncapped, routinely commissioned national PrEP programme across all SHCs in England commenced in October 2020 following the conclusion of the PrEP Impact Trial. Current guidelines on the use of PrEP developed by the British HIV Association/British Association for Sexual Health and HIV (BHIVA/BASHH) [12] recommend PrEP should be offered to HIV-negative MSM reporting recent (in the last six months) and ongoing condomless anal sex (CAS), including CAS with an HIV-positive partner not known to be virally suppressed. Proposed monitoring and evaluation frameworks [13, 14] recognise limitations in assessing PrEP-need and use in those not regularly engaging with sexual health services, highlighting the importance of supplementary, community monitoring.

The Gay Men’s Sexual Health Survey (GMSHS), a serial cross-sectional, self-administered survey in London commercial venues (e.g. clubs, pubs, bars, saunas), has provided regular, periodic monitoring of HIV/STI risk behaviours and HIV preventative behaviours in a community sample of MSM since 1996. The 2019 iteration of the GMSHS is the first to follow the start of the PrEP Impact Trial. Using data from GMSHS 2019, this analysis provides a description of PrEP use including unmet need in MSM attending London-based commercial venues in a period of widening PrEP availability.

## Methods

### Survey population and data collection

Survey methods have been previously described [15, 16], see Supplement 1 for 2019 survey. A total of 34 London venues primarily frequented by MSM agreed to take part. Venues and events were collated from previous survey listings as well as publications, and online forums. At participating venues, men aged ≥ 18 were asked to self-complete a sexual health questionnaire and, if willing, provide an oral fluid sample for anonymous HIV antibody (Ab) testing. Venues were visited up to three times from 8 June through 17 August 2019 by trained fieldworkers who provided study information and obtained verbal consent from study participants.

For analyses, MSM were defined as: self-identifying men (including trans men) who self-reported as gay, bisexual, demisexual, pansexual, or having had sex with a man in the last year. HIV status used for analyses was based on reported self-perception (deemed to be more influential on behaviour) [17, 18], or, where not specified, based on the result of last reported HIV test or the use of HIV antiretroviral treatment (ART).

### Oral fluid collection and HIV Ab testing

All study participants were asked to provide an oral fluid sample for HIV antibody testing using the Intercept i2heTM device (Orasure Technologies, Bethlehem, PA, USA). Fieldworkers provided guidance on self-collection and information where participants could obtain a named HIV test as sample testing was not diagnostic and results would not be disseminated to participants. Collected samples were stored anonymously in tamper-proof envelopes along with their associated questionnaire. Specimens were ambiently stored until transported to the National Infection Service (Public Health England, London, UK) for processing within a 21-day window. Testing and validation methods have been previously described [16].

HIV Ab results were examined by self-perceived HIV status. Undiagnosed HIV infections were defined in men with an HIV Ab positive result but reporting a self-perceived HIV-negative/unknown status. Oral fluid sample provision was investigated by self-perceived HIV status, age, and ethnicity using logistic regression, where univariate odds ratios (ORs) and 95% confidence intervals (CIs) were calculated. HIV Ab results were also examined in HIV-negative/unknown men reporting current PrEP use.

### Data management and statistical analysis

All survey data were double entered using Microsoft Access 2010. Any discordance was validated by a third reviewer. Data management and analyses were carried out using Stata v.15.0 (StataCorp, College Station, TX, USA).

### Self-reported PrEP use

Descriptive analyses of sociodemographic characteristics, service engagement and outcomes, as well as sexual risk and prevention behaviours were carried out in the wider survey population and in self-perceived HIV-negative/unknown MSM providing information on current PrEP use.

Sociodemographic characteristics examined included: age-group, ethnic group, region of birth, residence, employment status, and years of education since age 16. Service engagement and outcomes included the report of a SHC visit in the last year, recency of last HIV test, HIV and STI test frequency, and report of an STI diagnosis in the last year. Sexual risk behaviours included the report of CAS in the last three months, and the following in the last year: frequency of CAS partners, chemsex defined as the use of ketamine, gamma hydroxybutyrate (GHB)/gamma butyrolactone (GBL), mephedrone, and/or meth amphetamine before or during sex. Prevention behaviours included report of ever using HIV post-exposure prophylaxis (PEP), or the private or internet purchase of antibiotics for the prevention of STIs.

We also describe PrEP sourcing in HIV-negative/unknown MSM self-reporting PrEP use in the last year, and PrEP regimen in current users. PrEP sources in the last year were classified as: SHC (exclusive trial or non-trial), internet or private prescription, mixed (SHC and internet or private sourcing), and other (outside of SHC, internet or private sourcing).

### Current PrEP use in men with PrEP-need

Self-perceived HIV-negative/unknown MSM were considered to have ‘PrEP-need’ based on retrospective sexual risk and proxies based on available survey data. Using 2018 BHIVA/BASHH PrEP guidelines [12], PrEP-need was defined as the report of CAS in the last three months, or the report of CAS (in the last year) with an HIV-positive/unknown status partner not known to be on HIV treatment. This PrEP-need proxy utilised a conservative three-month look-back window, also used for PrEP Impact Trial eligibility, versus a six-month window recommended in BHIVA/BASHH guidance. Look-back windows for CAS with an HIV-positive/unknown status partner spanned a year due to question design. Men were considered to have unmet need when PrEP-need was identified and current PrEP use was not reported.

Multivariate logistic regression was used to examine associations between current PrEP use and sociodemographic characteristics (described above) in MSM with PrEP-need. Univariate associations were calculated, and characteristics were retained for multivariate models where p < 0.10. Evidence of association was considered where p < 0.05. Sequential, multivariate modelling examining associations between current PrEP use and service engagement and outcomes as well as sexual risk and prevention behaviours using multivariate logistic regression were also performed, where factors with univariate associations (p < 0.10) were adjusted for sociodemographic characteristics carried forward from prior models. Univariate odds ratios (ORs), adjusted odds ratios (aORs), 95% CIs, and associated *p*-values derived from the likelihood ratio test (LRT) were calculated.

Select service engagement and outcome variables used in modelling included: HIV test frequency in the last year, location of last HIV test (SHC or other), and report of an STI diagnosis in the last year. Sexual risk and prevention behaviours included report of the following in the last year: ≥ 5 CAS partners, ≥ 2 casual CAS partners (where a casual partner is defined as only having had sex with once), chemsex (described above), the private or internet purchase of antibiotics for the prevention of STIs, and ever reporting PEP use. All analyses were based on available information. Missing data imputation was not performed given limited denominator studies of MSM in London and the UK.

## Results

### Venues and survey population

Among the 2475 venue visitors approached, 1535 participated (62.0%); 127 were excluded (113 visitors that did not meet analysis inclusion and 14 MSM due to prior participation) leaving 1408 MSM for analysis (Supplement 2). The median age of self-perceived HIV-negative/unknown men (91.5%, 1288/1408) was 35 (interquartile range [IQR]: 28–44); median age in HIV-positive men (8.5%, 120/1408) was 45 (IQR: 37–51). Over three-quarters of participants were of white ethnicity (75.5% in HIV-negative unknown men, 75.8% in HIV-positive men). Further description of the community sample, stratified by self-perceived HIV status, is found in Table 1a.

**Table 1:** Sociodemographic characteristics, service engagement and outcomes, sexual risk and prevention behaviours in a) MSM by self-perceived HIV status and b) MSM providing information on current PrEP use, June to August 2019

	a) MSM by self-perceived HIV status^1,2^			b) MSM providing information on current PrEP use^1,2,3^		
	MSM participants	HIV negative/unknown	HIV positive	MSM participants	Current PrEP use	No current PrEP use
	*n* = 1408	*n* = 1288	*n* = 120	*n* = 1230	*n* = 242	*n* = 988
Recruitment location						
Bar/pub	72.9% (1026/1408)	72.6% (935/1288)	75.8% (91/120)	72.4% (891/1230)	70.7% (171/242)	72.9% (720/988)
Club	22.7% (320/1408)	23.1% (298/1288)	18.3% (22/120)	23.2% (28/1230)	24.0% (58/242)	23.0% (227/988)
Sauna	4.4% (62/1408)	4.3% (55/1288)	5.8% (7/120)	4.4% (54/1230)	5.4% (13/242)	4.2% (41/988)
Provided oral fluid sample						
No	38.3% (539/1408)	38.3% (493/1288)	38.3% (46/120)	37.5% (461/1230)	35.1% (85/242)	38.1% (376/988)
Yes	61.7% (869/1408)	61.7% (795/1288)	61.7% (74/120)	62.5% (769/1230)	64.9% (157/242)	61.9% (612/988)
HIV Ab result^5^						
Indeterminate	0.5% (4/855)	0.1% (1/784)	4.2% (3/71)	0.1% (1/758)	0.7% (1/153)	0.0% (0/605)
Negative	91.2% (780/855)	99.1% (777/784)	4.2% (3/71)	99.1% (751/758)	97.4% (149/153)	99.5% (602/605)
Positive	8.3% (71/855)	0.8% (6/784)	91.6% (65/71)	0.8% (6/758)	2.0% (3/153)	0.5% (3/605)
Sociodemographic characteristics						
Age						
Mean [Standard deviation]	37 [11.2]	37 [11.1]	44 [10.0]	37 [11.1]	35 [9.2]	37 [11.5]
Median [Interquartile range]	35 [29–45]	35 [28–44]	45 [37–51]	35 [28–44]	33 [28–41]	35 [29–45]
Age-group						
18–24	10.0% (139/1392)	10.8% (138/1276)	0.9% (1/116)	10.8% (132/1220)	8.7% (21/241)	11.3% (111/979)
25–34	37.6% (523/1392)	39.1% (499/1276)	20.7% (24/116)	38.5% (470/1220)	22.6% (106/241)	37.2% (364/979)
35–44	27.4% (381/1392)	27.3% (348/1276)	28.5% (33/116)	27.5% (336/1220)	35.3% (85/241)	25.6% (251/979)
≥ 45	25.1% (349/1392)	22.8% (291/1276)	50.0% (58/116)	23.1% (282/1220)	12.0% (29/241)	25.8% (253/979)
Ethnic group						
White	75.5% (1062/1406)	75.5% (971/1286)	75.8% (91/120)	75.5% (927/1228)	71.9% (174/242)	76.4% (753/986)
Black	4.1% (57/1406)	3.9% (50/1286)	5.8% (7/120)	3.9% (48/1228)	3.3% (8/242)	4.1% (40/986)
South East Asian	2.2% (31/1406)	2.4% (31/1286)	0.0% (0/120)	2.3% (28/1228)	2.9% (7/242)	2.1% (21/986)
Asian	4.3% (61/1406)	4.6% (59/1286)	1.7% (2/120)	4.6% (57/1228)	5.4% (13/242)	4.5% (44/986)
Latin American	4.9% (69/1406)	4.4% (57/1286)	10.0% (12/120)	4.5% (55/1228)	5.4% (13/242)	4.3% (42/986)
Mixed/Other	9.0% (126/1406)	9.2% (118/1286)	6.7% (8/120)	9.2% (113/1228)	11.2% (27/242)	8.7% (86/986)
UK-born						
No	45.6% (642/1386)	46.4% (589 /1269)	45.3% (53/117)	46.1% (559/1213)	49.6% (118/238)	45.2% (441/975)
Yes	53.7% (744/1386)	53.6% (680/1269)	54.7% (64/117)	53.9% (654/1213)	50.4% (120/238)	54.8% (534/975)
Residence						
London	81.0% (1133/1399)	80.2% (1026/1280)	89.9% (107/119)	80.1% (980/1223)	84.1% (201/239)	79.2% (779/984)
Outside London	11.6% (162/1399)	12.0% (153/1280)	7.6% (9/119)	12.0% (147/1223)	8.4% (20/239)	12.9% (127/984)
Outside UK	7.4% (104/1399)	7.9% (101/1280)	2.5% (3/119)	7.9% (96/1223)	7.5% (18/239)	7.9% (78/984)
Current employment						
No	12.6% (175/1393)	12.2% (155/1274)	16.8% (20/119)	12.0% (146/1218)	10.0% (24/240)	12.5% (122/978)
Yes	87.4% (1218/1393)	87.8% (1119/1274)	83.2% (99/119)	88.0% (1072/1218)	90.0% (216/240)	87.5% (856/978)
Education since age 16						
0–2 years	16.1% (222/1377)	15.6% (196/1260)	22.2% (26/117)	15.5% (187/1205)	10.9% (26/238)	16.7% (161/967)
≥ 2 years/still full-time	83.9% (1155/1377)	84.4% (1064/1260)	77.8% (91/117)	84.5% (1018/1205)	89.1% (212/238)	83.4% (806/967)
Service engagement and outcomes						
SHC visit in the last year						
No	30.0% (416/1389)	32.0% (406/1269)	8.3% (10/120)	32.2% (392/1216)	2.9% (7/241)	39.5% (385/975)
Yes	70.1% (973/1389)	68.0% (863/1269)	91.7% (110/120)	67.8% (824/1216)	97.1% (234/241)	60.5% (590/975)
Last HIV test						
Last 3 months	46.6% (642/1378)	47.0% (597/1269)	41.3% (45/109)	47.5% (577/1214)	88.8% (213/240)	37.4% (364/974)
Between 3–12 months ago	24.6% (339/1378)	25.7% (326/1269)	11.9% (13/109)	25.1% (305/1214)	7.9% (24/240)	28.9% (281/974)
More than a year ago	16.9% (233/1378)	17.8% (226/1269)	6.4% (7/109)	18.0% (218/1214)	0.8% (2/240)	22.2% (216/974)
Over 5 years ago	7.7% (106/1378)	5.0% (63/1269)	39.5% (43/109)	5.0% (60/1214)	0.4% (1/240)	6.1% (59/974)
Never	4.2% (58/1378)	4.5% (57/1269)	0.9% (1/109)	4.5% (54/1214)	0.0% (0/240)	5.5% (54/974)
≥ 4 HIV tests in the last year						
No	80.8% (996/1248)	79.1% (924/1168)	7.2% (72/80)	78.8% (879/1116)	42.0% (99/236)	88.6% (780/880)
Yes	20.2% (252/1248)	20.9% (244/1168)	10.0% (8/80)	21.2% (237/1116)	58.1% (137/236)	11.4% (100/880)
≥ 4 STI tests in the last year						
No	82.4% (1053/1278)	82.0% (956/1166)	86.6% (97/112)	81.8% (918/1123)	50.0% (116/232)	90.0% (802/891)
Yes	17.6% (225/1278)	18.0% (210/1166)	13.4% (15/112)	18.3% (205/1123)	50.0% (116/232)	10.0% (89/891)
STI diagnosis in the last year						
No	75.4% (1036/1375)	76.4% (960/1256)	63.9% (76/119)	76.5% (921/1204)	41.1% (99/241)	85.4% (822/963)
Yes	24.7% (339/1375)	23.6% (296/1256)	36.1% (43/119)	23.5% (283/1204)	58.9% (142/241)	14.6% (141/963)
Sexual risk and prevention behaviours						
CAS in the last 3 months						
No	48.5% (668/1378)	49.5% (625/1262)	37.1% (43/116)	49.7% (603/1213)	17.1% (41/240)	57.8% (562/973)
Yes	51.5% (710/1378)	50.5% (637/1262)	62.9% (73/116)	50.3% (610/1213)	82.9% (199/240)	42.2% (411/973)
≥ 5 CAS partners in the last year						
No	77.9% (968/1242)	79.4% (904/1138)	61.5% (64/104)	79.3% (867/1093)	39.9% (93/233)	90.0% (774/860)
Yes	22.1% (274/1242)	20.6% (234/1138)	38.5% (40/104)	20.7% (226/1093)	60.1% (140/233)	10.0% (86/860)
Chemsex in the last year^6^						
No	82.5% (1105/1340)	83.7% (1025/1225)	69.6% (80/115)	83.8% (988/1179)	66.0% (153/232)	88.2% (835/947)
Yes	17.5% (235/1340)	16.3% (200/1225)	30.4% (35/115)	16.2% (191/1179)	34.1% (79/232)	11.8% (112/947)
Ever used PEP						
No	83.8% (1156/1380)	83.8% (1062/1268)	83.9% (94/112)	83.8% (1023/1221)	58.6% (140/239)	89.9% (883/982)
Yes	16.3% (224/1380)	16.3% (206/1268)	16.1% (18/112)	16.2% (198/1221)	41.4% (99/239)	10.1% (99/982)
Purchase of antibiotics to prevent STIs^7^						
No	94.2% (1257/1335)	94.9% (1154/1216)	86.6% (103/119)	95.0% (1106/1165)	87.2% (204/234)	96.9% (902/931)
Yes	5.8% (78/1335)	5.1% (62/1216)	13.5% (16/119)	5.0% (59/1165)	12.8% (30/234)	3.1% (29/931)
PrEP use in the last year						
No	.	77.9% (954/1224)	.	78.1% (952/1219)	12.0% (29/241)	94.4% (923/978)
Yes	.	22.1% (270/1224)	.	21.9% (267/1219)	88.0% (212/241)	5.6% (55/978)
Current PrEP use						
No	.	80.3% (988/1230)	.	.	.	.
Yes	.	19.7% (242/1230)	.	.	.	.

^1^Self-identified men, including trans men, who self-reported as gay or bisexual, or who had sex with a man in the last year and did not previously participant in the survey in the last three months

^2^Based on self-perceived HIV status; where self-reported HIV status not specified, based on report of last HIV test as positive or antiretroviral medication use

^3^Self-perceived HIV-negative/unknown MSM

^4^Where information on current PrEP use was provided

^5^Where IgG > 0.200; excludes 14 samples

^6^Chemsex defined as self-reported use of ketamine, gamma hydroxybutyrate(GHB)/gamma butyrolactone(GBL), mephedrone and/or meth amphetamine before or during sex

^7^Private or internet purchase. PrEP = HIV pre-exposure prophylaxis. MSM = men who have sex with men. CAS = condomless anal sex. STI = sexually transmitted infection. *SHC* sexual health clinic

### HIV Ab testing

Of the 1408 MSM included in descriptive analyses, 869 (61.7%) MSM provided an oral fluid sample; 855/869 (98.4%) had immunoglobulin G (IgG) levels suitable for HIV Ab testing. Of these, 8.3% (71/855) were HIV Ab positive, and 0.47% (4/855) indeterminate. Undiagnosed HIV infection was identified in 8.5% (6/71) of MSM who had a positive HIV Ab result and reported a self-perceived HIV negative/unknown status (Table 1a). Among MSM with a self-perceived HIV-positive status, 91.6% (65/71) had a concordant HIV Ab result, while 4.2% (3/71) were HIV Ab negative and 4.2% (3/71) had an indeterminate result (Table 1a).

Oral fluid provision was not associated with self-perceived HIV status (OR: 1.00, 95% CI: 0.68–1.47; 61.7% in both HIV-negative/unknown and HIV-positive men) (Table 1a). However, men of black ethnicity were less likely to provide a sample versus men of white ethnicity (36.8% vs 63.8%; OR: 0.33, 95% CI: 0.19–0.58). Men aged ≥ 35 were less likely to provide a sample compared to those aged < 35 (58.9% vs 65.3%; OR: 0.76, 95% CI: 0.61–0.95).

### PrEP use in the last year and sourcing

**Fig. 1:**
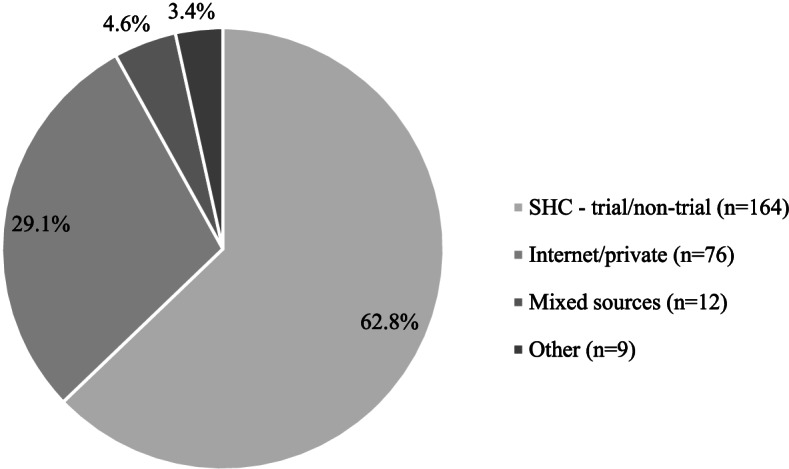
PrEP sourcing in self-perceived HIV-negative/unknown MSM reporting PrEP use in the last year^1^

PrEP use in the last year was reported in 22.1% (270/1224) of MSM who were self-perceived HIV-negative/unknown (Table 1a). Most reported sourcing from a SHC (62.8%, 164/261), with nearly one-third reporting sourcing from internet/private prescription (29.1%, 76/261) (Fig. 1).

### Current PrEP use and regimen

Current PrEP use was reported in 19.7% (242/1230) of men who were self-perceived HIV-negative/unknown. Median age of current users was 33 (IQR: 28–41), most were of white ethnicity (71.9%, 172/242), and 89.0% (212/238) reported higher education levels since age 16 (≥ 2 years/still in full-time education). Four in five users reported CAS in the last three months (82.9%; 199/240) and over half reported ≥ 4 HIV tests (58.1%; 137/236) in the last year (Table 1b). Further description of MSM reporting current PrEP use is found in Table 1b.

In men specifying current PrEP regimen, 76.5% (176/230) reported daily use with 75.8% (125/165) of users reporting complete adherence in the last two weeks. In event-based users (23.5%; 54/230), 66.0% (35/53) reported their most recent PrEP dose in the last two weeks.

Among the 758 MSM who were self-perceived HIV-negative/unknown and who provided an oral fluid sample (Table 1b), 153 men reported current PrEP use, of whom three had an HIV Ab positive result (2.0%). Of these, two men reported daily dosing with complete adherence in the last two weeks, while one event-based user reported their most recent dose more than two weeks prior; all reported engaging in CAS and having had an HIV test in the last three months. In current non-users, HIV Ab positivity was 0.5% (3/605), where one of three men had identified PrEP-need [0.35% (1/284) HIV Ab positivity in non-users with PrEP-need, not shown].

### Unmet PrEP-need

Of the 1230 self-perceived HIV-negative/unknown men who provided information on current PrEP use, 632 (51.4%) met PrEP-need proxy measures (610 of whom reported CAS in the last three months) (Supplement 2). Over two-thirds of this group (68.2%; 431/632) did not report PrEP use. 41 men reporting current PrEP use (41/242, 16.9%) did not meet the PrEP-need proxies and were not included in PrEP-use analyses.

In MSM with identified PrEP-need, current PrEP use was associated with age (men aged < 25 less likely to report PrEP use than older men) as well as education (men reporting higher levels of education more likely to report PrEP use) (Table 2). In multivariate modelling, there were slight decreases in effect measures for age-group (aOR: 3.52, 95% CI: 1.76–7.02 in men aged 40–44 vs men aged 18–25) and education (aOR: 1.72, 95% CI: 1.01–2.92 in men with ≥ 2 years/still in full-time education vs no or < 2 years of education since age 16), however, evidence of association to current PrEP use persisted.

**Table 2:** Select sociodemographic characteristics associated with self-reported current PrEP use in self-perceived HIV-negative/unknown MSM with identified PrEP-need, June to August 2019

	MSM with identified PrEP-need^a,b^		OR (95% CI)	*p* value^c^	aOR^d^ (95% CI)	*p* value^c^
	Current PrEP use	No current PrEP use				
	*n* = 201	*n* = 431				
Sociodemographic characteristics						
Age-group (years)						
18–24	8.0% (16/201)	15.0% (64/428)	1.00 (ref)	0.001	1.00 (ref)	0.003
25–29	24.4% (49/201)	21.5% (92/428)	2.13 (1.11–4.07)		2.07 (1.08–3.97)	
30–34	20.4% (41/201)	22.4% (96/428)	1.71 (0.88–3.30)		1.71 (0.88–3.32)	
35–39	14.4% (29/201)	11.7% (50/428)	2.32 (1.14–4.74)		2.29 (1.12–4.69)	
40–44	20.9% (42/201)	11.0% (47/428)	3.57 (1.80–7.11)		3.52 (1.76–7.02)	
≥ 45	11.9% (24/201)	18.5% (79/428)	1.22 (0.60–2.48)		1.25 (0.61–2.56)	
Ethnic group						
White	75.1% (151/201)	77.5% (334/431)	1.00 (ref)	0.513	.	.
Ethnic minority	24.9% (50/201)	22.5% (97/431)	1.14 (0.77–1.69)			
UK-born						
No	50.8% (101/199)	46.1% (197/427)	1.00 (ref)	0.282	.	.
Yes	49.3% (98/199)	53.9% (230/427)	0.83 (0.59–1.16)			
UK resident						
No	7.0% (14/199)	6.8% (29/429)	1.00 (ref)	0.899	.	.
Yes	93.0% (185/199)	93.2% (400/429)	0.96 (0.49–1.86)			
Current employment						
No	8.5% (17/200)	11.6% (50/431)	1.00 (ref)	0.231	.	.
Yes	91.5% (183/200)	88.4% (381/431)	1.41 (0.79–2.52)			
Education since age 16						
None/up to 2 years	10.6% (21/199)	17.3% (73/423)	1.00 (ref)	0.025	1.00 (ref)	0.040
≥ 2 years/still full-time	89.5% (178/199)	82.7% (350/423)	1.78 (1.05–2.97)		1.72 (1.01–2.92)	

^a^Based on 632 self-perceived HIV negative/unknown MSM who provided information on current PrEP use and had identified PrEP-need defined as self-reported CAS in the last three months and/or CAS with a HIV-positive/unknown status partner not known to be on ART in the last year; 31.8% (201/632) of men reported current PrEP use

^b^Analyses exclude 41 current PrEP users that did not have identified PrEP-need

^c^Likelihood ratio test (LRT) *p* value

^d^Adjusted for age-group and education since age 16; 619 observations in adjusted model. *OR* odds ratio, *aOR* adjusted odds ratio, PrEP = HIV pre-exposure prophylaxis. *CAS* condomless anal sex, Ethnic minority = men of black, south east Asian, Asian, Latin American and mixed/other ethnic groups

Current PrEP users reported higher levels of service engagement, sexual risk and prevention behaviours, as well having had a prior STI diagnosis, compared to non-users with PrEP-need (Table 3). Adjusting for age-group and education, current users were more likely in the last year to have reported: ≥ 4 HIV tests (aOR: 9.68, 6.35–14.8), last HIV test at a SHC (aOR: 5.28, 95% CI: 2.97–9.36), an STI diagnosis (aOR: 8.46, 95% CI: 5.69–12.6), ≥ 5 CAS partners (aOR: 10.1, 95% CI: 6.71–15.2), ≥ 2 casual CAS partners (aOR: 9.21, 95% CI: 6.03–14.1), chemsex (aOR: 2.85 95% CI: 1.90–4.27), the purchase of antibiotics to prevent STIs (aOR: 4.58, 95%: 2.33–9.02), and to have ever used PEP (aOR: 4.95, 95%: 3.30–7.41).

**Table 3:** Service engagement and outcomes, and sexual risk and prevention behaviours associated with self-reported current PrEP use in self-perceived HIV-negative/unknown MSM with identified PrEP-need, June to August 2019

	MSM with identified PrEP-need^a,b^		OR (95% CI)	*p* value^c^	aOR^d^ (95% CI)	*p* value^c^
	Current PrEP use	No current PrEP use				
	*n* = 201	*n* = 431				
Service engagement and outcomes						
≥ 4 HIV tests in the last year						
No	41.5% (83/200)	86.9% (351/404)	1.00 (ref)	< 0.001	1.00 (ref)	< 0.001
Yes	58.5% (117/200)	13.1% (53/404)	9.34 (6.24–14.0)		9.68 (6.35–14.8)	
Location of last HIV test						
Other	8.0% (16/200)	30.3% (125/413)	1.00 (ref)	< 0.001	1.00 (ref)	< 0.001
SHC	92.0% (184/200)	69.7% (288/413)	4.99 (2.87–8.67)		5.28 (2.97–9.36)	
STI diagnosis in the last year						
No	35.8% (72/201)	80.8% (344/426)	1.00 (ref)	< 0.001	1.00 (ref)	< 0.001
Yes	64.2% (129/201)	19.3% (82/426)	7.52 (5.16–10.9)		8.46 (5.69–12.6)	
Sexual risk and prevention behaviours						
≥ 5 CAS partners in the last year						
No	33.3% (66/198)	81.8% (342/418)	1.00 (ref)	< 0.001	1.00 (ref)	< 0.001
Yes	66.7% (132/198)	18.2% (76/418)	9.00 (6.11–13.2)		10.1 (6.71–15.2)	
≥ 2 casual CAS partners in the last year						
No	24.6% (46/187)	73.0% (267/366)	1.00 (ref)	< 0.001	1.00 (ref)	< 0.001
Yes	75.4% (141/187)	27.1% (99/366)	8.27 (5.51–12.4)		9.21 (6.03–14.1)	
Chemsex in the last year^e^						
No	62.9% (122/194)	82.1% (339/413)	1.00 (ref)	< 0.001	1.00 (ref)	< 0.001
Yes	37.1% (72/194)	17.9% (74/413)	2.70 (1.84–4.00)		2.85 (1.90–4.27)	
Purchase of antibiotics to prevent STIs^f^						
No	86.3% (170/197)	96.4% (397/412)	1.00 (ref)	< 0.001	1.00 (ref)	< 0.001
Yes	13.7% (27/197)	3.6% (15/412)	4.20 (2.18–8.10)		4.58 (2.33–9.02)	
Ever used PEP						
No	55.8% (111/199)	86.5% (370/428)	1.00 (ref)	< 0.001	1.00 (ref)	< 0.001
Yes	44.2% (88/199)	13.6% (58/428)	5.06 (3.41–7.50)		4.95 (3.30–7.41)	

^a^Based on 632 self-perceived HIV negative/unknown MSM who provided information on current PrEP use and had identified PrEP-need defined as self-reported CAS in the last three months and/or CAS with a HIV-positive/unknown status partner not known to be on ART in the last year; 31.8% (201/632) of men reported current PrEP use

^b^Analyses exclude 41 current PrEP users that did not have identified PrEP-need

^c^Likelihood ratio test (LRT) p value

^d^Adjusted for age-group and education since age 16

^e^Chemsex defined as self-reported use of ketamine, gamma hydroxybutyrate (GHB)/gamma butyrolactone (GBL), mephedrone and/or meth amphetamine before or during sex

^f^Private or internet purchase. *OR* odds ratio, *aOR* adjusted odds ratio, PrEP = HIV pre-exposure prophylaxis, PEP = HIV post-exposure prophylaxis, *SHC* sexual health clinic, *STI* sexually transmitted infection, *CAS* condomless anal sex, *SHC* sexual health clinic

## Discussion

Overall, 2019 GMSHS results provide a snapshot of PrEP use and behaviour in a community sample of MSM prior to national PrEP programme implementation and before the impact of Covid-19. Positively, the use of PrEP has risen substantially since 2016 [16] but considerable unmet need was found, highlighting potential access barriers against the backdrop of falling national HIV incidence and increasing STI rates among MSM.

Despite low coverage across men with identified PrEP-need, current PrEP users reported high levels of behaviours associated with greater HIV/STI risk and prior HIV/STI prevention behaviours. Though the cross-sectional design limits insight on the sequence of reported risk behaviours and current PrEP use, these findings correspond with guidance recommendations [12] and suggest successful targeting to those with greatest need. However, behavior change and risk compensation as a result of PrEP use cannot be discounted in this study.

Though most men were found to engage with SHCs, high PrEP-need and HIV/STI risk reporting in non-users suggests that PrEP access may have been limited. During the time of this survey, release of additional PrEP Impact Trial places for London were in negotiation following recruitment pauses for MSM across several London SHCs [11]. This may have contributed to PrEP access barriers and influenced sourcing outside of SHCs. Previously described PrEP initiation and persistence barriers [19] may also have affected uptake.

Encouragingly, PrEP use in men with identified need was not associated with ethnicity, however, larger studies to assess equity by ethnicity in those with PrEP-need will be required given potential for sampling bias. Moreover, the survey sample size limited examination by individual ethnic groups who, in descriptive analyses had disparate PrEP use. While longitudinal evidence suggests increasing PrEP awareness in SHC-attending MSM in England since 2013 [20], user inequalities in populations at greatest need for PrEP should be closely monitored given slower rates of HIV diagnosis declines in black and other minority ethnic populations in the UK [1]. Results do suggest user disparity by age and education as older age-groups and men reporting higher levels of education were more likely to have reported current PrEP use. Similar age-related differences in PrEP initiation and uptake have been reported [20–22]. As the national PrEP programme in England continues, user inequalities in MSM accessing PrEP should continue to be closely monitored, and include community insights through peripheral periodic monitoring and outreach.

In our sample, a third of MSM described PrEP sourcing outside of SHCs. Shifts to SHC-sourced PrEP will likely increase demand on publicly funded service provision, as users also reported regular HIV testing and STI diagnoses in the last year. With greater PrEP availability through a national programme, high STI rates on PrEP initiation and potential increases to STI diagnoses ascertained through regular testing or as a result of risk compensation [5, 23–26], will require additional clinical resource. Among all reporting current PrEP use, most men described daily, adherent use; though report of adherence is encouraging, further validation studies are needed to inform clinical practice and user education needs. Additional assessment of user knowledge and understanding of PrEP stop-and-restart, as well as regimen switching [27], both not assessed in this survey, are required as evidence of event-based efficacy increases [6, 28].

As a whole, outcomes from this 2019 community sample mirror described national HIV and STI trends [1, 2]; undiagnosed HIV infection in MSM remained low relative to GMSHS 2016 [16] [8.5% (6/71) in 2019 from 13.2% (5/38) in 2016], while men reporting an STI diagnosis in the last year increased [24.7% (339/1375) in 2019 from 19.8% (147/743) in 2016]. High levels of recent HIV testing persisted, as most men reported an HIV test in the last year [71.2% (981/1378) in 2019 from 69.7% (514/738) in 2016], affirming continued HIV testing engagement seen in national surveillance.

HIV Ab antibody negativity found in self-perceived HIV-positive men highlights a potential reduction of sensitivity among ART users [29] but was not a focus of this analysis. While HIV Ab results indicate positivity in some PrEP users, results should be interpreted with caution as both PrEP use and perceived HIV status were self-reported; for PrEP users, vigorous monitoring of baseline and incident infection remains important given known diagnostic challenges and possible seroconversion delay [30, 31]. HIV treatment resistance in PrEP users has been reported [32, 33] and monitoring following widespread PrEP scale-up has been recommended [34].

Despite sustained levels of reported HIV/STI risk behaviours relative to GMSHS 2016, there was parallel reporting of STI and HIV preventative behaviours. Interestingly, one in ten current PrEP users reported private or internet antibiotic purchase for STI prevention. This, along with similar UK survey findings [35], signals the need for additional management considerations in PrEP care and the importance of continued antibiotic resistance surveillance of bacterial STIs as MSM continue to report self-procured STI prophylaxis against wider national advice [36].

### Limitations

Men attending venues may not be representative of the wider London MSM population. Venues did include longstanding establishments frequented by HIV outreach where venue visitors may have been more aware of HIV prevention options including PrEP. Due to the cross-sectional design, we have limited insight to the temporality of reported risks or behaviours and PrEP outcomes; however, factors examined in multivariate analyses likely preceded the current PrEP-use outcome. We cannot establish whether reported risks or behaviours followed PrEP initiation, but irrespective of sequence, most PrEP users had identified PrEP-need. Responses reported in relation to a recall period may be subject to recall bias. Further, social desirability bias may have affected PrEP reporting, given previously reported social norms and experienced stigma described by PROUD participants [37]; however, survey anonymity should have limited this. Non-response varied across questions, however, the wider survey population and those providing a response to PrEP outcomes are broadly similar; PrEP-need proxies for men reporting CAS (in the last year) with an HIV-positive/unknown status partner not on HIV treatment may have been underestimated as a result of non-response in question subsets. As retrospective risk may not reflect current risk, need may be overestimated, but can inform an upper limit based on prior risk. Recent inclusion of behavioural risk indicators in national STI surveillance should refine PrEP-need estimates in MSM accessing PrEP through SHCs in England [38, 39]. Due to venue age-limits, men aged < 18 were not included in this study, limiting age-related interpretation in men with PrEP-need, however, results do reflect reported age disparities in PrEP initiation.

## Conclusion

Though PrEP use in MSM has increased, coverage was low in men with PrEP-need. PrEP use may, however, have been influenced by capped and transient access during the summer of 2019. Reported self-sourcing provides insight to additional access demands to a national programme as well as service and outreach considerations for men that may choose to access PrEP outside of SHCs. PrEP appears to be appropriately indicated for men in this sample given use in men reporting high proportions of HIV/STI risk behaviours and prior HIV/STI prevention behaviours, however, further examination of non-use in men with PrEP-need in the context of an uncapped national programme is warranted.

Key challenges to England’s nationally commissioned PrEP programme will include addressing unmet need and equitable PrEP uptake, as well as ensuring sustained service engagement and access to combination prevention service pathways, especially in those continuing to source PrEP outside of sexual health services. Equally, user messaging encouraging regular HIV testing using robust diagnostics is imperative to limit undetected baseline or incident HIV infection. These findings should be considered for programme, intervention, and outreach planning in order to address the suggested PrEP user inequalities among younger men with lower educational attainment, as England’s national PrEP programme moves through its first year.

Given extensive PrEP sourcing outside of sexual health services, continued community surveillance, as a component of programme monitoring and evaluation framework, is essential to assess appropriate targeting and equitable access to all those that could most benefit from PrEP. Despite anticipated shifts [14] from private sourcing to that from a commissioned programme, service disruptions caused by Covid-19 related lockdowns may delay this transition as the pandemic continues.

As PrEP awareness and use expands, its effect on HIV incidence will become more pronounced. Increasing uptake, in younger men who may benefit from PrEP could accelerate already substantial declines in HIV incidence. Outreach and PrEP access and uptake interventions should target this key population for continued prevention gains. As England aims for zero new HIV transmissions, investment in equitable and sustained PrEP access, especially in those not regularly engaging with sexual health services, must remain a priority.

## Supplementary Information

**Additional file 1: Supplement 1. **Gay Men’s Sexual Health Survey (GMSHS) 2019. **Supplement 2. **Gay Men’s Sexual Health Survey 2019 analysis flow chart.

## Data Availability

Study data from this analysis are available with support from the study sponsor and with a data sharing agreement in place. Requests can be directed to the corresponding author.

## Abbreviations

BASHH: British Association for Sexual Health and HIV
BHIVA: British HIV Association
CAS: Condomless anal sex
GMSHS: Gay Men’s Sexual Health Survey
MSM: Gay, bisexual, and other men who have sex with men
PEP: HIV post-exposure prophylaxis
PrEP: HIV pre-exposure prophylaxis
SHC: Sexual health clinic
STI: Sexually transmitted infection
UK: United Kingdom
