# Supplementary material for: PrEP use and unmet PrEP-need among men who have sex with men in London prior to the implementation of a national PrEP programme, a cross-sectional study from June to August 2019
Source: BMC Public Health. 2022 Jun 3;22:1105. doi: 10.1186/s12889-022-13425-0 (PMC9163522; doi:10.1186/s12889-022-13425-0)
Supplement: Supplementary file 1 — Additional file 1: Supplement 1. Gay Men’s Sexual Health Survey (GMSHS) 2019. Supplement 2. Gay Men’s Sexual Health Survey 2019 analysis flow chart. [file 12889_2022_13425_MOESM1_ESM.docx]

**Supplement 1. Gay Men’s Sexual Health Survey (GMSHS) 2019**


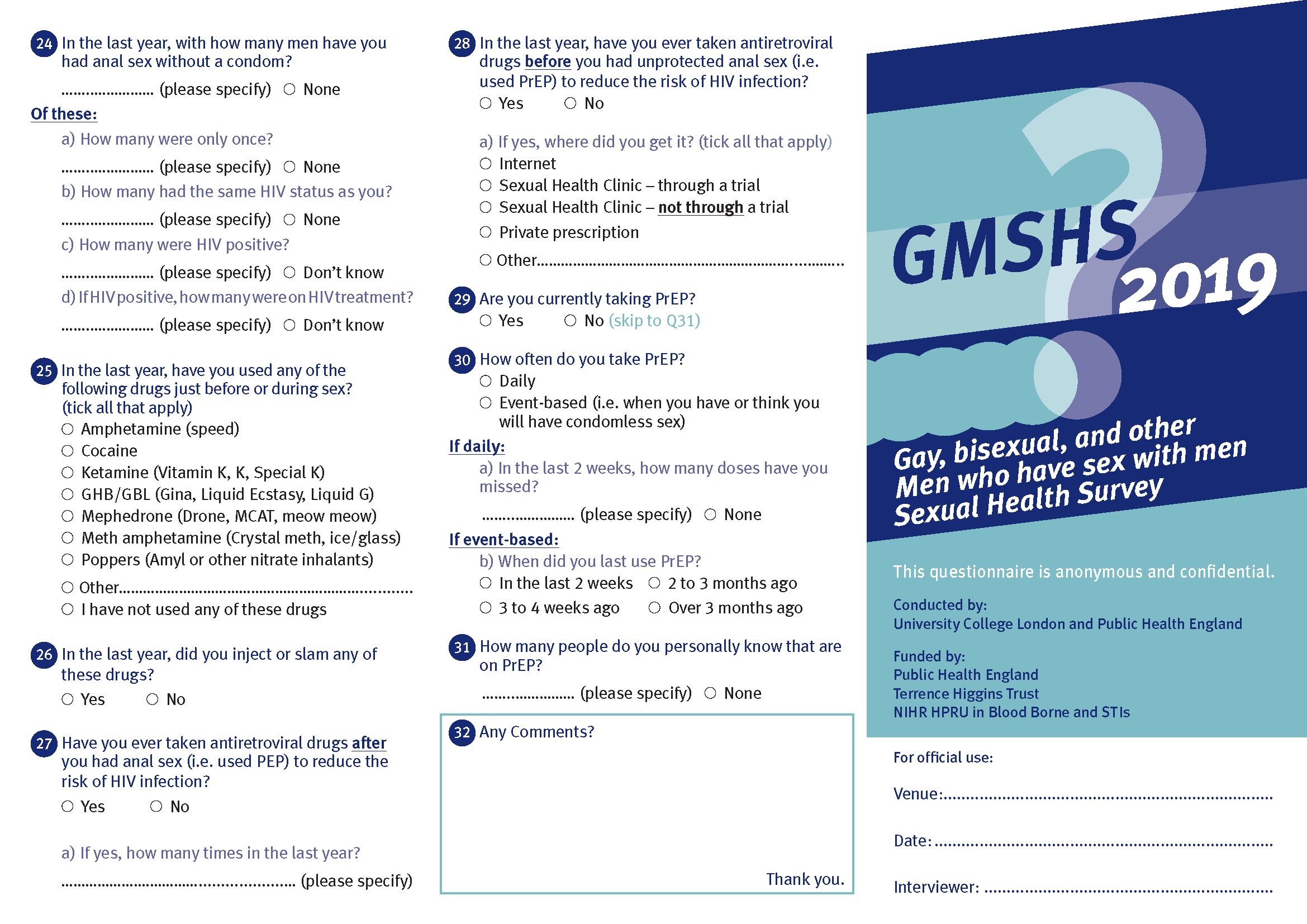


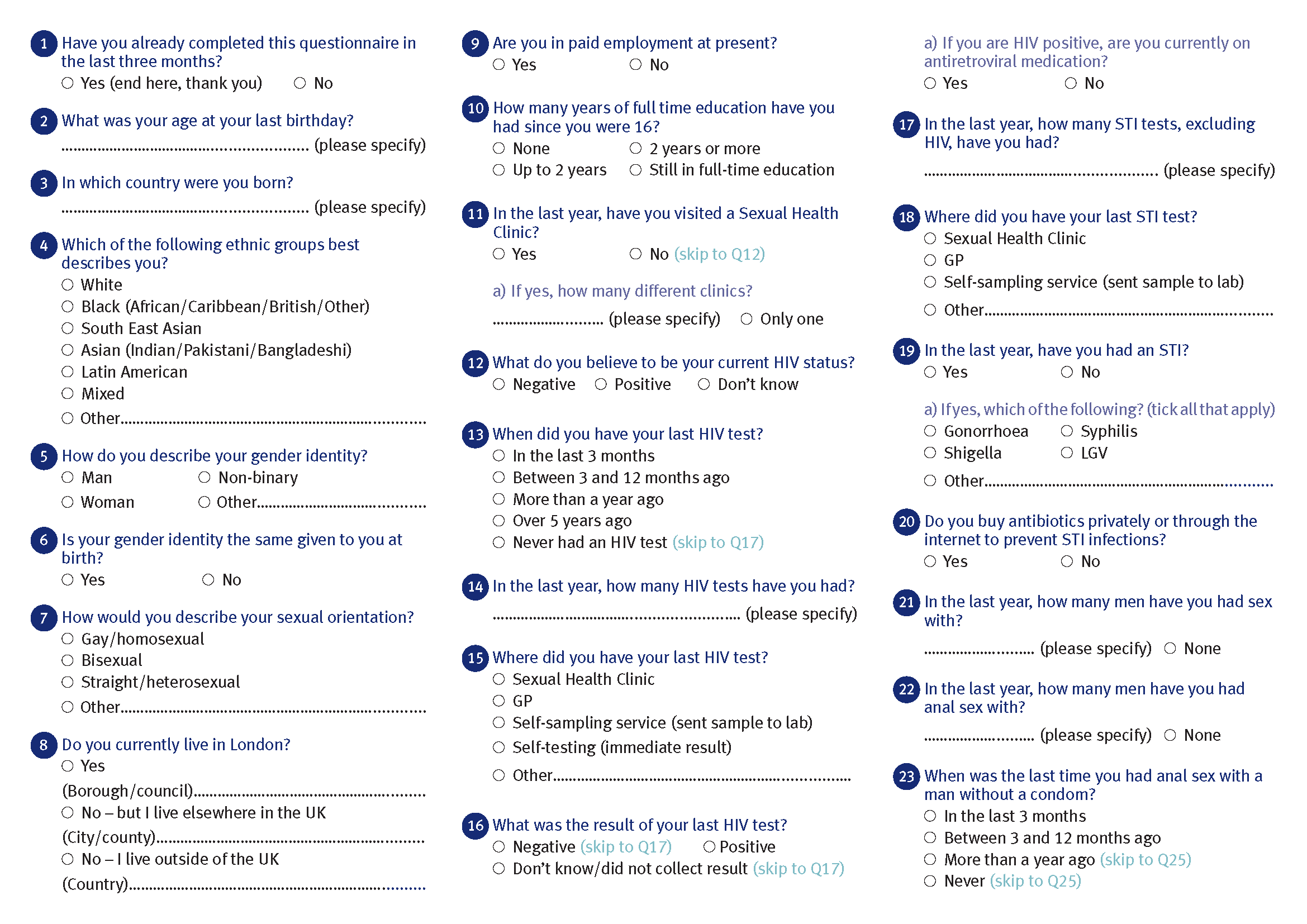


**Supplement 2. Gay Men’s Sexual Health Survey 2019 analysis flow chart.**


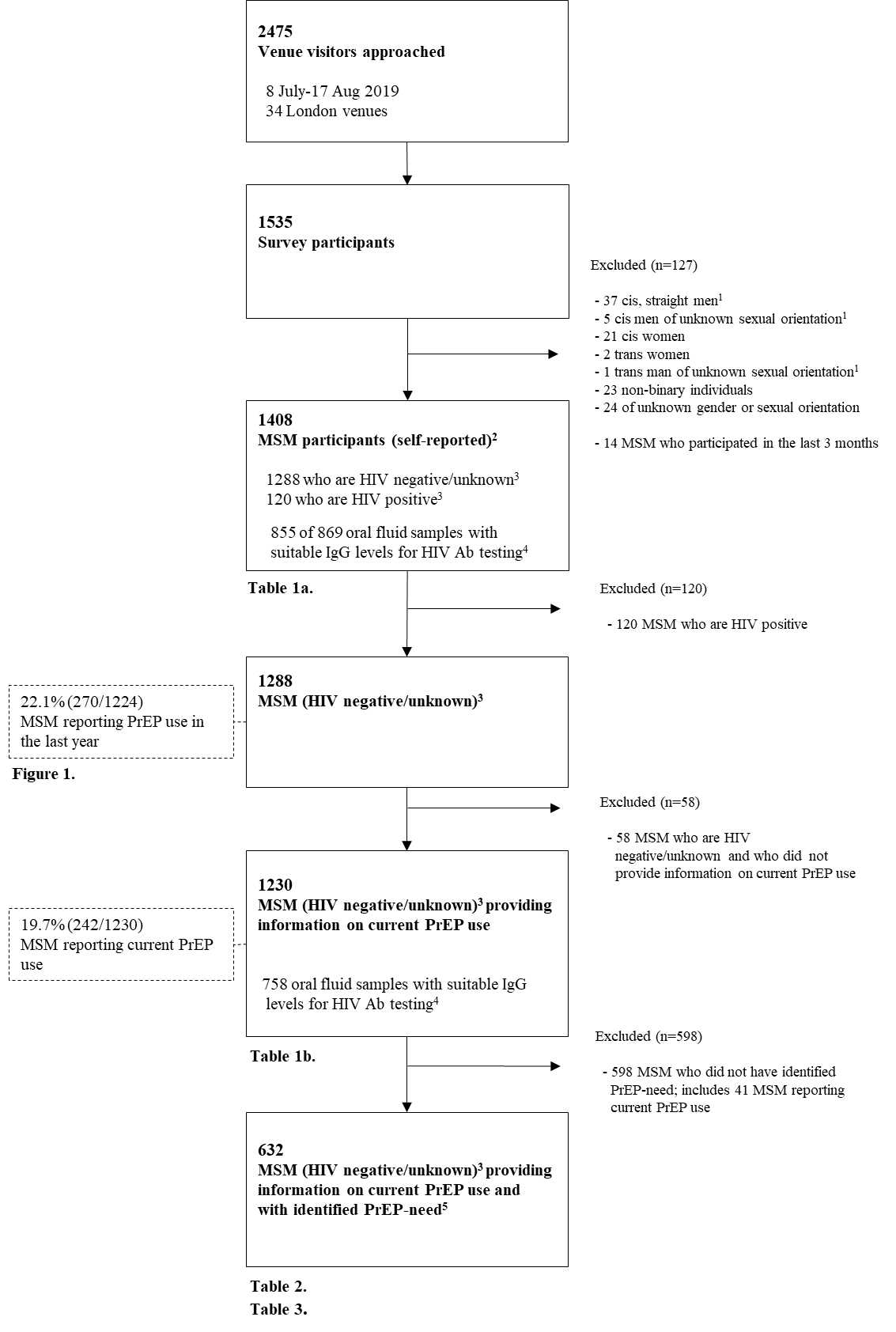


^1^ Did not report sex with a man in the last year. ^2^ Self-identified men, including trans men, who self-reported as gay or bisexual, or who had sex with a man in the last year and did not previously participant in the survey in the last three months. ^3^ Based on self-perceived HIV status. ^4^ Where IgG>0.200; excluded 14 total samples. ^5^ PrEP-need defined as self-reported CAS in the last three months and/or CAS with a HIV positive/unknown status partner not known to be on ART in the last year. PrEP=HIV pre-exposure prophylaxis. MSM=men who have sex with men. CAS=condomless anal sex. ART=antiretroviral treatment.
